# Vicariance and Its Impact on the Molecular Ecology of a Chinese Ranid Frog Species-Complex (*Odorrana schmackeri*, Ranidae)

**DOI:** 10.1371/journal.pone.0138757

**Published:** 2015-09-22

**Authors:** Yongmin Li, Xiaoyou Wu, Huabin Zhang, Peng Yan, Hui Xue, Xiaobing Wu

**Affiliations:** 1 Anhui Province Key Laboratory for Conservation and Exploitation of Biological Resource, College of Life Sciences, Anhui Normal University, Wuhu, Anhui, China; 2 School of Life Sciences, Fuyang Teachers College, Fuyang, Anhui, China; University of Innsbruck, AUSTRIA

## Abstract

Paleogeological events and Pleistocene climatic fluctuations have had profound influences on the genetic patterns and phylogeographic structure of species in southern China. In this study, we investigated the population genetic structure and Phylogeography of the *Odorrana schmackeri* species complex, mountain stream-dwelling odorous frogs, endemic to southern China. We obtained mitochondrial sequences (1,151bp) of the complete ND2 gene and two flanking tRNAs of 511 individuals from 25 sites for phylogeographic analyses. Phylogenetic reconstruction revealed seven divergent evolutionary lineages, with mean pairwise (K2P) sequence distances from 7.8% to 21.1%, except for a closer ND2 distance (3.4%). The complex geological history of southern China drove matrilineal divergence in the *O*. *schmackeri* species complex into highly structured geographical units. The first divergence between lineage A+B and other lineages (C-G) had likely been influenced by the uplift of coastal mountains of Southeast China during the Mio-Pliocene period. The subsequent divergences between the lineages C-G may have followed the formation of the Three Gorges and the intensification of the East Asian summer monsoon during the late Pliocene and early Pleistocene. Demographic analyses indicated that major lineages A and C have been experienced recent population expansion (c. 0.045–0.245 Ma) from multiple refugia prior to the Last Glacial Maximum (LGM). Molecular analysis suggest that these seven lineages may represent seven different species, three described species and four cryptic species and should at least be separated into seven management units corresponding to these seven geographic lineages for conservation.

## Introduction

Past geological events and climatic oscillations have played important roles in forming the contemporary genetic diversity and population structure of animals across the globe [1–3]. The relative roles played by geography and climate in driving genetic patterns, have important implications for speciation and diversification [1]. Southern China, an area containing a mosaic of mountains such as Nanling, Wuyi, Huangshan and Tianmu Mts., harbours high levels of species diversity [4–6]. Previous studies on other co-distributed anurans demonstrated that the coastal mountains were one of the causes of lineage divergence [7–9]. The orogenesis of the mountains in southeastern China may have driven the formation of lineage divergence of *O*. *schmackeri*, a mid-to-low altitude range frog species.

Uplifting of the Qinghai-Tibet Plateau (QTP) rearranged the drainage systems and these geological events may have influenced the geographic patterns and genetic structure of species [10]. During the Pliocene, the upper reaches of the Yangtze River formed a tributary of the paleo-Red River flowing southwards into the South China Sea [11] and was kept isolated from the middle drainage system at the Three Gorges. Subsequently, in the late Pliocene and early Pleistocene, the rapid uplifts of the eastern QTP rearranged the drainage systems of the Yangtze River and contributed to the connection of the watercourses at the Three Gorges [12,13]. Moreover, the accelerating uplift had a decisive effect on the formation and strengthening of the East-Asia monsoon [12,14]. Both drainage evolution and climate have profoundly influenced the genetic diversity and demographic history of organisms in this region [5,15,16]. Thus, the drainage rearrangement of the Yangtze River may have been one among the important driving forces for the determination of the current genetic structure of the *O*. *schmackeri* species complex.

Global cyclical cooling-warming events during the Quaternary have resulted in periodic expansions and contractions of population size and distribution range of species [17]. For example, during Pleistocene glaciations, Northwest Europe and the most northern regions in North America were covered with ice sheets [1,2]. Consequently, in Europe and America, species retreated to southern refugia during glacial episodes and then expanded northward again during interglacial periods [18–23]. In contrast, the glacial advance in East Asia was not as extensive as in Europe and North America, and southern China experienced a relatively mild Pleistocene climate [24]. Thus, climatic cycling might not have led to population shrinkages in southern China [7,8,25,26].

China is known to have hosted many important global Pleistocene refugia for lineages that evolved prior to the late Tertiary and Quaternary glaciations [27]. Most of previous phylogeographic studies elucidating the complex role of past climate change and geology on geographical distribution range and demographic history, mainly focused on taxa from QTP and adjacent mountain ranges [28–35], from Yungui Plateau [36–39], and from Qinling-Daba Moutains region [40,41]. Recent studies on herpetological, avian and mammal phylogeography evaluated the effects of Pleistocene climatic changes and geological accidents on diversification of populations in southern China [7–10,25,26,42–47]. To our knowledge, there is no record on other vertebrates’ phylogeographic studies in this region. The scarcity of data limits our understanding of spatial distribution, lineage sorting, and historical demography associated with Pleistocene climatic oscillations and geological events in southern China.

The Chinese piebald odorous frog (*Odorrana schmackeri*), belonging to the Ranidae family, is widely distributed and occurs in southern and south-central China [48,49]. *O*. *schmackeri* is characterized by clearly visible brown or black round spots on the head and back. It is a sexually dimorphic species, with males and females measuring about 44mm and 80mm respectively, in snout-vent length. The species is higly adapted to mountain environments, inhabiting the moist evergreen broad-leaf forests at 200-1400m altitude [48]. Its wide distribution and strict habitat requirements make it an excellent model to study the effects of the past geological events and climatic cycles on the genetic structure and demographic history in this region. Owing to its wide distribution area, *O*. *schmackeri* is variable in body size, skin color and some other morphological traits. In recent years, new cryptic species have been discovered and named: *O*. *yizhangensis* occurs in Mangshan of Yizhang County, Hunan Province, at 1000–1200 m altitude, and in Nanling of Ruyuan county, Guangdong Province, at 900–1100 m elevation; *O*. *huanggangensis* occurs in Wuyi Mts., Fujian and eastern Jiangxi provinces, at 200–800 m elevation; *O*. *tianmuii* occurs in Tianmu Mts., northern Zhejiang, at 200–800 m [49]. In order to test whether the recently recognized species represent distinct evolutionary lineages, we included their populations in this study and call them collectively the *O*. *schmackeri* species complex.

The present study investigated the phylogeography of *O*. *schmackeri *species complex based on samples collected from almost the entire distribution range of *O*. *schmackeri*, *O*. *yizhangensis*, *O*. *huanggangensis* and *O*. *tianmuii*, using mtDNA sequence of 1,151bp. We examined the following three hypotheses: (1) the orogenesis of the mountains in southeastern China drove the formation of lineage divergence of *O*. *schmackeri* species complex; (2) the drainage rearrangement of the Yangtze River was important driving force to shape the current genetic structure of *O*. *schmackeri* species complex; (3) lineages experienced recent population expansion prior to LGM.

## Materials and Methods

### Ethics statement

The capture, measurement, toe clip, and release procedures of the 511 Chinese piebald odorous frogs were approved by the Chinese Wildlife Management Authority which had the right to consent to such investigation work. At the same time, this study was approved by the Ethics Committee of Anhui Normal University and conducted following the Law of People’s Republic of China on the Protection of Wildlife. A part of the sampling was carried out in protected areas and approved by the Wildlife Management Stations of Daiyunshan National Nature Reserve, Nanling National Nature Reserve and Guniujiang National Nature Reserve. The most of sampling was conducted outside protected areas whithout any requirements for specific permission. According to the regulation for the Collection of Genetic Resources (HJ 628–2011), all frogs were captured by hand, then measured for morphometric characters. Toe-clip tissues (up to 1 cm length) were collected and preserved in 95% ethanol immediately after removal; live frogs were subsequently released at the capture point after treating wounds with antiseptic.

### Sampling of specimens

**Fig 1:**
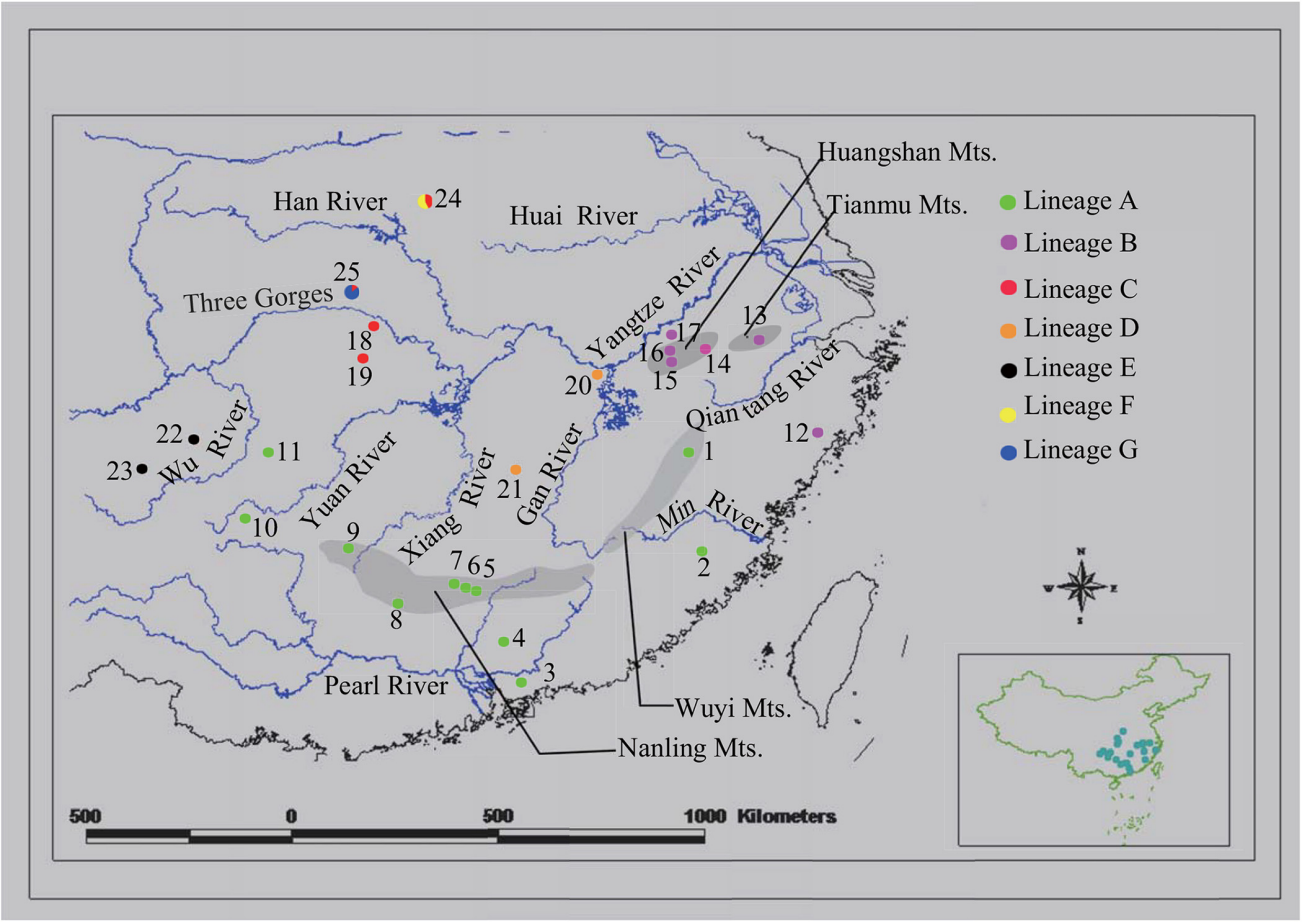
The geographic distribution of *O*. *schmackeri* species complex sampled in this study. Localities are detailed in S1 Table. Populations are presented as pie-diagrams with slice-size proportional to the frequency of the major lineages. Colors of pie-diagrams correspond to the lineages in Figs 2 and 3.

We analyzed a total of 511 individuals from 25 localities covering most of the distribution range of *O*. *schmackeri* species complex (S1 Table; Fig 1). Six individuals of *O*. *margaratae* from Shennongjia Mts. (SNJ) and one individual of *O*. *graminea* from Huangshan Mts. (HS) were used as outgroups based on the results of Chen et al. [50].

### DNA extraction, PCR amplification and sequencing

Tissue samples were digested using proteinase K, and total DNA was subsequently extracted following the standard phenol/chloroform method [51]. A 1151 base pairs (bp) fragment of mitochondrial DNA containing the complete mitochondrial NADH dehydrogenase subunit 2 (ND2) gene and two flanking transfer RNA (tRNA) genes was amplified with the primer pair Ile-LND2 (5’-ATAGGGAGACTTATAGGGGTTC-3’) and Asn-HND2 (5’-CTAAGTCATTACGGGATCGAGGCC-3’) designed according to the complete mitochondrial genome of *O*. *schmackeri* from Li et al. [52] (GenBank accession no. KJ149452). The 30μl PCR reaction volume contained 10×reaction Buffer 3μl, 25mmol/L MgCl_2_ 2μl, 2mmol/L dNTPs 2μl, 10μmol/L each primer 1μl, 1 unit of Taq DNA polymerase (TaKaRa Biotechnology (Dalian) Co., Ltd., Dalian, China), and 20–60 ng template DNA. PCR procedure consisted of an initial denaturation at 94°C for 5 min, then 32 cycles with 94°C for 40 s, 60°C for 40 s and 72°C for 50 s, followed by a final extension at 72°C for 10 min. The PCR products were separated by electrophoresis in 1.5% agarose gels, then were purified by DNA Gel Extraction Kit (V-gene, Hangzhou, China) with ABI 3730 (Shanghai Sangon Biotechnology Co., Ltd., Shanghai, China). The DNA products were purified, and then sequenced in both directions on an ABI3730 with an ABI PRISM BigDye terminator Cycle Sequencing Ready Reaction Kit (Perkin-Elmer Biosystems).

### Sequences alignment and phylogenetic analyses

Sequences were edited and aligned manually using BIOEDIT version 7.0.9.0 [53], and the protein coding region ND2 was translated into amino acids for confirmation of alignment.

Haplotypes for ND2+tRNA sequences were identified using the program DnaSP 5.10 [54]. Bayesian inference (BI) analysis was employed to construct the phylogeny. Log-likelihood scores were obtained using PAUP* 4.0b10 [55] and used to conduct the testing of evolution models by Akaike information criterion (AIC) in the MODELTEST 3.7 [56]. The Bayesian analysis was performed with MrBayes 3.2 [57] based on the best-fit substitution model (GTR+I+G). Four independent runs were performed for 10 million generations and trees were sampled every 1,000th generation resulting 10,000 trees. The stationarity of the likelihood scores of sampled trees was determined in Tracer 1.6 [58]. The first 25% were discarded as burn-in, and the remaining trees were used to estimate Bayesian posterior probabilities.

### Divergence time estimation

To estimate divergence time, we used an uncorrelated lognormal relaxed molecular clock model as implemented in BEAST v1.75 [59] based only on the entire ND2 sequences. We were only interested in the divergence times of the major clades, therefore, we simplified the data set for the BEAST analysis. Most similar haplotypes of *O*. *schmackeri* species complex were excluded, and only twenty haplotypes represented the major lineages were included. Thirteen additional taxa were introduced to provide useful calibration points: *O*. *graminea* (KP167578), *O*. *margaratae* (KP167579), *O*. *ishikawae* (AB511282), *Rana dybowskii* (KF898355), *R*. *chensinensis* (KF898356), *R*. *temporaria* (AF314018), *R*. *boylii* (AF314019), *R*. *sylvatica* (AF314017), *Pelophylax plancyi* (EF196679), *P*. *nigromaculata* (AB043889), *P*. *cretensis* (GU812136), *P*. *bedriagae* (GU812075), *P*. *lessonae* (JN627426).

Divergence age estimates were established in this study for *Pelophylax cretensis* and *Pelophylax bedriagae* (log-normal distribution with youngest of 5 Ma and standard deviation [SD] of 0.159) based on geological data (5–5.5 Ma) [60]. Divergence between *Rana sylvatica* and *Rana boylii* (31.2 Ma, 8.1SD) was estimated based on a multiple gene/calibration analysis [61].

We applied a GTR+I+G model of evolution based on MODELTEST 3.7, and a Yule process for speciation. Three independent MCMC runs were conducted for 10 million generations, each with a burn-in of 1 million generations, and sampled every 1,000 generations. These three runs were then combined in Tracer 1.6 to determine burn-in and convergence of the chains.

### Population genetic analysis

We calculated the number of haplotypes (*h*), haplotype diversity (*Hd*) and nucleotide diversity (*π*) for both overall and each population using the program DnaSP 5.10. To investigate the level of genetic variation between populations, analyses of molecular variance (AMOVA) [62] were performed with 1,000 permutations in ARLEQUIN 3.5 [63]. We also calculated pairwise *F*_*ST*_ values among all the geographical populations using ARLEQUIN 3.5. Divergence between the matrilines was estimated by Kimura’s two-parameter (K2P) model [64] as implemented in MEGA 6 [65].

To examine the hypothesis of isolation by distance (IBD), the correlation between pairwise genetic (*F*_*ST*_ values) and geographical distance was tested and the significance level was estimated using Mantel permutation procedures in TFPGA 1.3 [66].

A haplotype network was constructed using TCS 1.21 [67] with a 95% connection limit followed. Loops in the resulting network were resolved following the criteria summarized by Pfenninger & Posada [68]. The network was nested by hand, following the rules outlined in Templeton et al. [69–71].

### Population demographic history

Tajima’s *D* [72] and Fu’s *Fs* [73] statistics were calculated and used to infer historic population expansion in DnaSP 5.10 with 10,000 bootstrap simulations. Mismatch distributions were used to detect population expansion [74] by using ARLEQUIN 3.5. The sum of square deviations (SSD) and raggedness index (RI) between the observed and the expected mismatch were used as a test statistic under a null hypothesis of a sudden population expansion. *P*-values were calculated as the probability of simulations producing a greater value than the observed value with 1000 bootstrap replicates.

If the sudden expansion model was not rejected, the time of population expansions (*t*, time in generations) was estimated using the formula *τ* = 2*ut* [74], where *τ* was the mode of mismatch distribution, and *u* was the mutation rate per generation for the entire sequence under study. The value of *u* was calculated using the equation *u* = *μk*, where μ was the mutation rate per nucleotide per generation and k was the number of nucleotides assayed.

## Results

### Authenticity of mitochondrial DNA

The fragment examined included three segments: the complete mitochondrial NADH dehydrogenase subunit 2 (ND2) gene and the flanking transfer RNA (tRNA) genes tRNA-Met (partial), and tRNA-Trp (entire) (hereafter referred to as ND2+tRNA).

All of ND2 sequences (complete length 1,032bp or 1,035bp) successfully translated to amino acids without premature stop codons, and the complete tRNA-Trp had stable secondary structures, indicating that the sequences were obtained from functional genes. Moreover, light strand sequences showed an expected bias against guanine (G = 10.8%, A = 30.2%, T = 28.8%, and C = 30.2%). These data indicated that only the mitochondrial genes were sequenced.

### Phylogenetic reconstruction

Of the entire 1,151 base pairs (bp) of aligned ingroup nucleotides, 359 sites were variable and 348 sites were parsimony informative. There were 3 sites with alignment gaps or missing data. We identified 94 unique haplotypes among 511 *O*. *schmackeri* species complex ND2+tRNA sequences (S2 Table). The sequences were deposited in GenBank under accession numbers (KP167484-KP167577).

**Fig 2:**
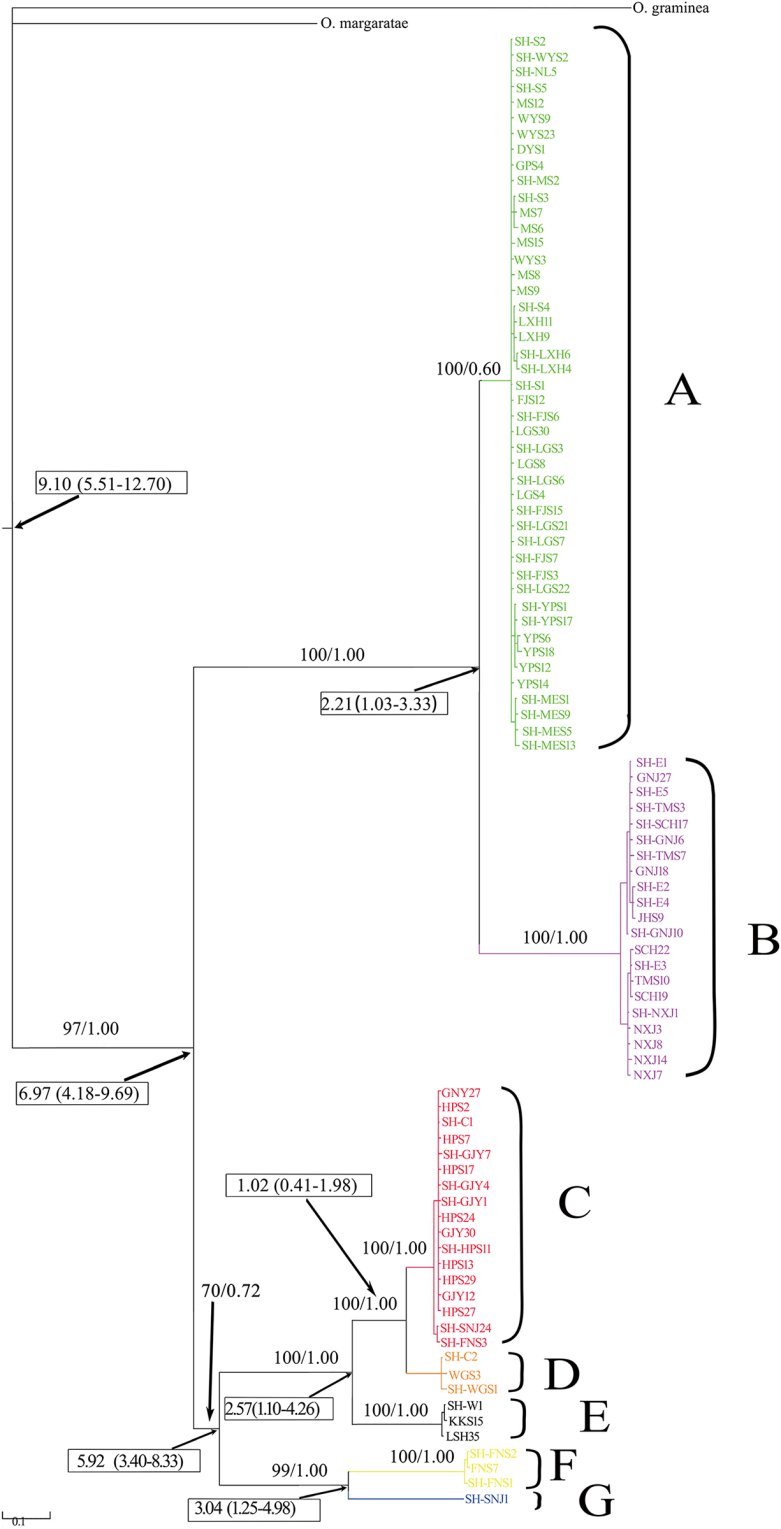
The Bayesian phylogenetic tree of *O*. *schmackeri* species complex based on the ND2+tRNA sequences. Numbers above branches represent Bayesian posterior probability (PP). The estimated divergence times with a 95% HPD between the major clades are given in rectangular boxes.

BI tree revealed that *O*. *schmackeri* species complex was composed of seven geographically structured clades (clades A, B, C, D, E, F and G in Fig 2). Clade A was characterized by having the largest number of haplotypes (46), occupying a large distribution region in South China, and forming a southern group. Also Clade B had a broad distribution, ranging from Anhui Prov. to Zhejiang Prov. in East China, and formed a separated eastern group. Clade C consisted of fifteen haplotypes from Gaojiayan and Hupingshan Mts. and two haplotypes from Shennongjia and Funiushan Mts., and formed a central group. Clade D was composed of two populations from Lushan and Wugongshan Mts. Clade E (hereafter termed the southwestern group) only contained samples from a narrow region of the northwestern Guizhou. Three Funiushan Mts. (FNS) haplotypes and one Shennongjia Mts. (SNJ) haplotype formed Clade F and Clade G, respectively.

Clade A was associated with the Pearl and Min Rivers. Clade B was distributed mainly in the basin of the Qiantang River, with some individuals occurring in the Yangtze River. Finally, the other five clades (Clades C-G) were broadly distributed in the middle and down tributaries of the Yangtze River.

### Genetic diversity and structure

Only thirteen out of 94 haplotypes (13.83%) generated by 511 *O*. *schmackeri* species complex ND2+tRNA sequences were shared among different sampling sites from same regional group. Eighty-one (86.17%) were unique haplotypes which were restricted to a single sampling locality and we found no shared haplotype among different regional groups (S2 Table).

Hierachical analyses of molecular variance (AMOVA) revealed significant values (P < 0.001) of among-group variance (*F*_*CT*_) when numbers of population groups were assumed to be 3, 5 or 7. The seven groups (A-G) were recognized as the most parsimonious geographic subdivisions because the grouping resulted in the highest values of among-group variance (*F*_*CT*_ = 0.98429, P < 0.001) (Table 1; Figs 1 and 2).

**Table 1:** Analysis of molecular variance (AMOVA) among ND2+tRNA sequences of O. schmackeri species complex.

Source of variation	Variance	% Total	Fixation indices	*P* value
Among groups	74.45503	98.43	*F*_*CT*_ = 0.98429	<0.0001
Among populations within groups	0.76201	1.01	*F*_*SC*_ = 0.64107	<0.0001
Within populations	0.42664	0.56	*F*_*ST*_ = 0.99436	<0.0001

Diversity indices, *Hd* and *π*, are summarized in S1 Table and Table 2. Among all individuals, both haplotype diversity (*Hd* = 0.945 ± 0.004) and nucleotide diversity (*π* = 0.10087 ± 0.00882) showed high values (S1 Table). Variation of haplotype diversity among populations was considerable, ranging from 0 to 0.883 ± 0.061 (S1 Table). Similarly, the values of nucleotide diversity ranged from 0 to 0.07740 ± 0.02104.

**Table 2:** Summary statistics observed in major lineages of *O*. *schmackeri* species complex.

Lineage	*N*	*h* /*Hd*	*π*	Tajima’s *D*	Fu’s *Fs*	SSD * (*P*_SSD_)	Raggedness index (*P*_Rag_)	*tau* *	Expansion time (Ma)
A	199	46/0.888	0.00215	-2.28730**	-80.486**	0.00505 (0.16)	0.03567 (0.37)	2.695	0.245
B	111	21/0.618	0.00256	-0.76300	-19.261**	0.04109 (0.50)	0.05099 (0.77)	9.555	-
C	71	17/0.644	0.00099	-2.20992**	-22.841**	0.07493** (0.00)	0.04332 (1.00)	0.516	0.045
D	33	3/0.273	0.00073	-	-	0.09883 (0.03)	0.66965 (0.49)	3.000	-
E	57	3/0.070	0.00006	-	-	0.00003 (0.25)	0.74690 (0.76)	3.000	-
F	6	3/0.733	0.00122	-	-	0.03396 (0.53)	0.13333 (0.76)	2.037	-

* SSD sum of squared deviation, *tau* expansion parameter

** *P*<0.01

As for genetic diversity among lineages, because of the small number of populations and the few haplotypes found in the lineages D-G, analyses of genetic diversity focused only on lineages A, B and C, which were represented by 46, 21 and 17 haplotypes, respectively. Lineage A had high haplotype diversity and relatively low nucleotide diversity, while Lineage B possessed lowest haplotype diversity and relatively high nucleotide diversity. Given the relatively small numbers of the sample size and the sampling site, Lineage C possessed lower haplotype diversity and extremely low nucleotide diversity (Table 2). Genetic divergence ranged from 3.4% to 21.1% between lineages A-G (Table 3).

**Table 3:** Mean genetic distances among different lineages of *O*. *schmackeri* species complex based on the Kimura 2-parameter model (lower-left), standard error (upper-right) estimated by bootstrap method (replication = 1000).

Lineage	A	B	C	D	E	F	G
A		0.008	0.014	0.014	0.013	0.014	0.014
B	0.079		0.015	0.016	0.015	0.015	0.016
C	0.175	0.204		0.005	0.009	0.013	0.013
D	0.176	0.206	0.034		0.008	0.014	0.013
E	0.174	0.201	0.078	0.078		0.012	0.013
F	0.175	0.196	0.164	0.164	0.157		0.010
G	0.188	0.211	0.150	0.156	0.155	0.103	

A Mantel test revealed that the correlation between genetic distance (*F*_*ST*_) and geographical distance was not significant (*r* = 0.4888, *P* = 0.9990), indicating that the genetic differentiation within *O*. *schmackeri* species complex did not fit the IBD model.

### Divergence dates

The estimated divergence times between the major lineages were mapped on the BI tree (Fig 2). The estimated TMRCA of the entire ingroup was 9.10 Ma (Late Miocene) with a 95% HPD of (5.51, 12.70). The estimated divergence times among lineages were from Late Miocene to Early Pleistocene. The earliest split within *O*. *schmackeri* species complex was estimated at 6.97 Ma (95% HPD, 4.18–9.69 Ma) during Late Miocene to Early pliocene. The divergence time between Lineage A and Lineage B was estimated to be 2.21 Ma (95% HPD, 1.03–3.33 Ma) and lineages C-G diverged from 1.02 to 5.92 Ma (95% HPD, 0.41–1.98 to 3.40–8.33 Ma).

### Haplotype network

**Fig 3:**
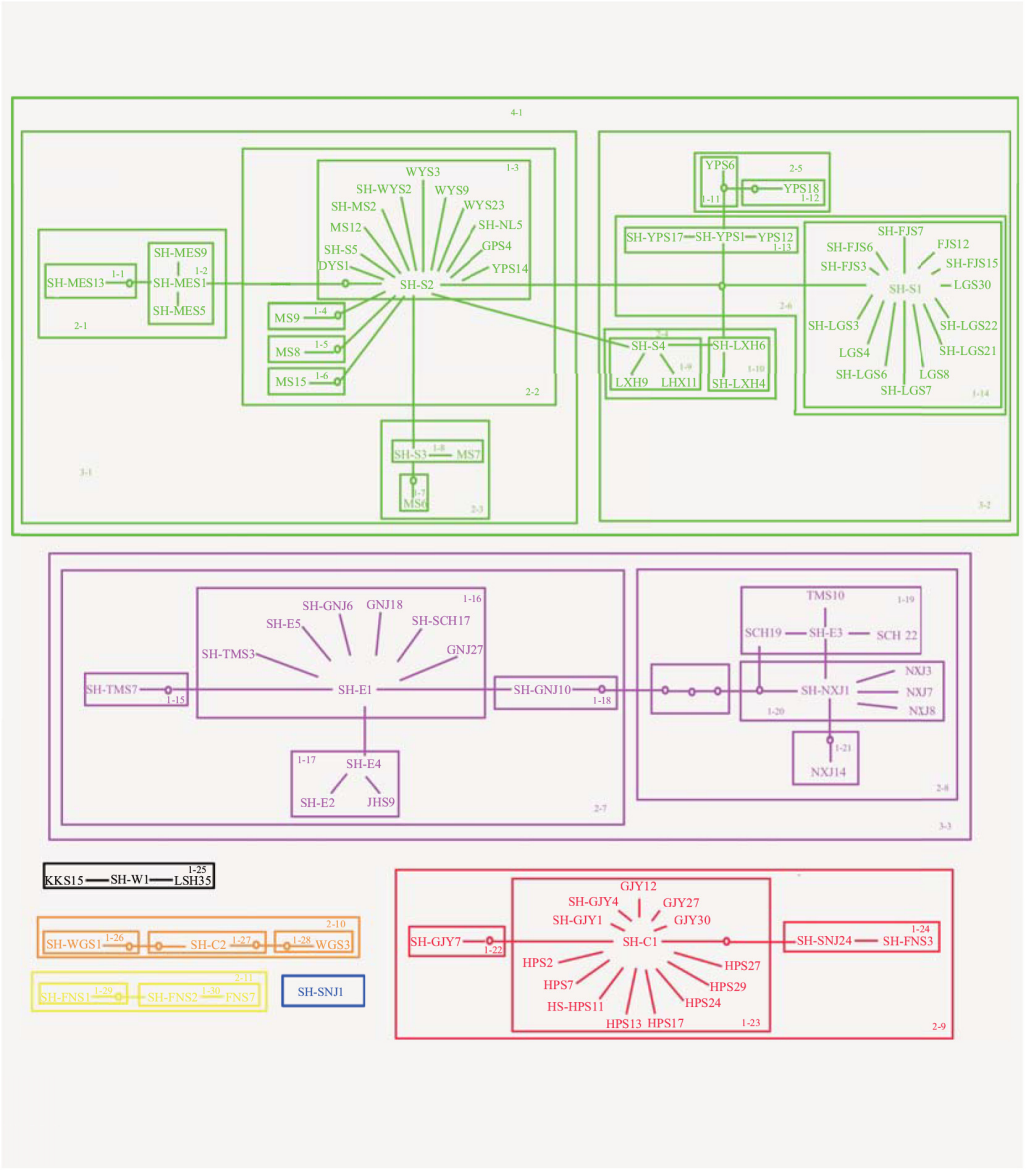
Haplotype networks and nested clade design for *O*. *schmackeri* species complex. Colours represent different lineages. The haplotype codes correspond to S2 Table. Unsampled haplotypes were represented by small closed circles.

The statistical parsimony network was constructed for O. schmackeri species complex with haplotypes connected by six or less mutational steps at a 95% confidence level. The rule resulted in six separate networks, corresponding to the six clades A-F in the BI tree, as well as one separate single haplotype (SH-SNJ1) which was concordant with the clade G (Fig 3). In cladogram 4–1 (Lineage A), 46 haplotypes formed two typical star-shape subnetworks connected by a missing haplotype. Two haplotypes (SH-S1 and SH-S2) occupied central positions, from which many other relative haplotypes could have originated through one or two steps of mutation. Except for the five shared haplotypes (SH-S1-S5), most haplotypes were observed in only a single location, indicating highly genetic differentiation among geographical populations. The nested design revealed 14 first-level clades, six-second-level clades, two-third-level clades and the total cladogram.

In cladogram 3–3 (Lineage B), 21 haplotypes connected in the network formed a more diffuse structure, containing 7-one-step clades, 2-two-clades and the total cladogram. Two haplotypes (SH-E1 and SH- E3) showed a widely geographical distribution in East China.

In cladogram 2–9 (Lineage C), 17 haplotypes generated a typical star-shape network, and a shared haplotype (SH-C1), located in the central position, was implied to be the ancestral haplotype.

### Demographic history

**Fig 4:**
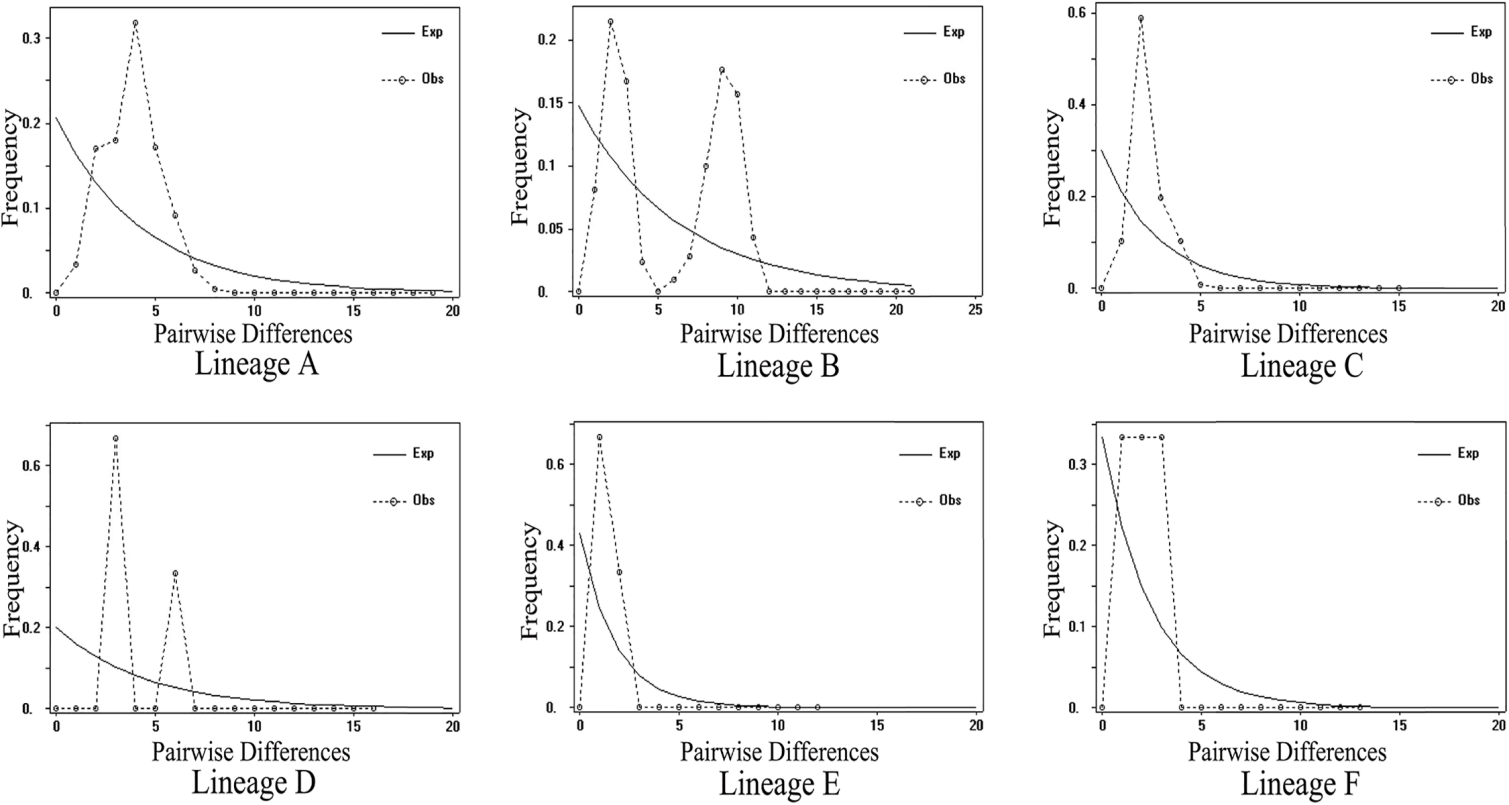
Mismatch distributions for six major lineages of *O*. *schmackeri* species complex. The dotted and thin lines represent the observed and expected mismatch distributions of a stationary population, respectively.

Unimodal distribution, indicative of demographic expansion, was observed for both lineages A and C (Fig 4), and raggedness indices suggested that the observed distributions did not significantly differ from the distributions expected under a model of sudden population expansion (*P* > 0.05, Table 2). In neutrality tests, the lineages A and C showed significantly negative values for Fu's *Fs* and Tajima’s *D* statistics (*P* < 0.01), also indicating that the lineages A and C had undergone a sudden demographic expansion. Based on the estimated mean rate of 0.957% [75] substitutions per site per million years, we estimated the demographic expansion time of lineages A, C to have occurred c. 0.245 and 0.045 Ma, respectively (Table 2).

## Discussion

### Lineage divergence

All analyses unambiguously showed that *O*. *schmackeri* species complex was genetically structured. About 86% of the total haplotypes occurred just in one location. No haplotypes were shared among all the 25 populations of *O*. *schmackeri* species complex. The AMOVA analysis also revealed a high level of geographic structuring. Moreover, there was no unambiguous link among the six haplotype networks and the separate haplotype (SH-SNJ1). The results showed significant genetic differentiation among the species complex.

The results of this study showed seven main mitochondrial haplotype lineages (A-G) within *O*. *schmackeri* species complex, roughly corresponding to seven regional groups (Fig 1). The seven lineages presented 3.4% to 21.1% mtDNA difference. The high levels of differences reflect long-term isolation, which is likely associated with the complex geological history of southern China. The first divergence between lineage A+B, mainly occurring in the Pearl, Min and Qiantang Rivers and other lineages (C-G) occupying midstream and downstream tributaries of the Yangtze River was estimated during the late Miocene approximately at 6.97 Ma (95% HPD, 4.18–9.69 Ma). The coastal mountains of Southeast China, such as Naling, Wuyi, Huangshan and Tianmu Mts. seem to have acted as the boundary between lineage A+B and the remaining lineages. The orogenesis of the mountains in southeastern China was estimated to have begun in the Middle Jurassic [76], and during the Mio-Pliocene period the mountains were further uplifted to approximately 1000m, thus forming a natural barrier separating the lineages between the southeast Coastal range and Yangtze River basin [77]. The coastal mountains, such as Nanling, Wuyi, Huang and Tianmu Mts. have been previously speculated to be one of the causes of lineage divergence of other anurans [7–9]. Similar effects of mountains on population isolation and lineage divergence have also been reported in other vertebrates. In China, the existence of Qinling Mts. has been speculated to be one of the causes of lineage divergence of the ring-necked pheasant (*Phasianus colchicus*) [78] and an endemic Chinese gecko, *Gekko swinhonis* [79].

Drainage evolution have been revealed as an important driving force for shaping current geographic patterns of stream-associated amphibians [10,35,36]. Historically, the upper tributaries of Yangtze River flowed southwards into the paleo-Red River [11,80], the middle reaches of Yangtze River were composed of inland rivers and lakes and lower Yangtze River flowed into the East China Sea [80]. During the Pliocene the upper Yangtze River drainage was isolated from the middle drainage system at the Three Gorges. The rapid uplifts of the eastern QTP rearranged the drainage systems of the Yangtze River and strengthened the East Asian summer monsoon during the late Pliocene and early Pleistocene at about 3.6±2.6Ma, when the Yangtze River shifted its drainage network and the upper and middle Yangtze River unified in the Three Gorges [12,13]. The estimate time (1.02–5.92Ma) of divergences among the lineages C-G occurring in the midstream and downstream tributaries of the Yangtze River occurred approximately during this period. When summer monsoons underwent substantial intensification, precipitation increased due to strengthened circulation which caused the Yangtze River and its major tributaries to become wider and to increase their flow rates [14]. Therefore, the subsequent divergences between the lineages C-G occupying the midstream and downstream tributaries of the Yangtze River may have followed the formation of the Three Gorges and the strengthening of the East Asian summer monsoon. It is reasonable to assume that the Yangtze River and its major tributaries such as Gan, Xiang, Yuan, Wu and Han Rivers formed natural barriers separating the lineages occupying the Yangtze River basin. Similar effects of rivers on population isolation and lineage divergence have also been reported in other anurans [36,40,81].

### Multiple glacial refugia

Suitable habitats and relatively stable microclimates in refugia may permit species to persist for long periods of time and even to speciate. Unique genotypes and substantial genetic diversity often occur in these locations [2]. According to the coalescent theory, the most frequent and widespread haplotype which occupies a central position in the haplotypes network is expected to be ancestral haplotype [71]. The geographical distribution and genealogical divergences are consistent with the scenario of multiple refugia. We proposed that there have been five independent refugia during Pleistocene glaciations. Several recent studies supported the eastern monsoon region and the lower elevations of the southwestern plateau as glaciation refugia [25,32,44,45]. In this study, the existence of seven mitochondrial lineages, with high haplotype and nucleotide diversity suggest five relict refugia for *O*. *schmackeri* species complex. Within lineage A, MS has substantial genetic diversity, a large number of private haplotypes, and the ancestral haplotype (SH-S2) is present, suggesting that the Nanling Mts. have serviced as a glacial refugium for this lineage. Similarly, Huangshan, Hupingshan, Wugongshan Mts. and northwestern Guizhou Plateau, might have represented the main refugia for lineage B, C, D, E, respectively.

Some refugia proposed by our study are corroborated by other case studies; for example, Hupingshan Mts. was considered as a refugium for the three sharp cedar *Cephalotaxus oliveri* [82], Huangshan Mts. was a refugium for the stout newts of genus *Pachytriton* [47], and Nanling Mts. was a refugium for the canopy tree *Eurycorymbus cavaleriei* [83]. More examples, as herein, are needed to support generalizations regarding the impacts of glacial oscillations on genetic and demographic legacies of species in southern China.

### Demographic history

The smooth and unimodal mismatch distributions, significantly negative Fu's *Fs* and Tajima’s *D* and the patterns of genetic diversity reported here strongly supported the idea of recent expansion of the lineages A and C.

However, unlike temperate species in North America and Europe which commonly expanded in the post-Last Glacial Maximum (LGM) era [84,85], the time since demographical expansion in the lineages A and C was estimated around 0.045–0.245 Ma, earlier than the LGM, and similar to the patterns observed for vertebrate species with wider distribution in East Asia that experienced population growth before the LGM [7,8,25,26,44,78,79]. This was likely due to the development of monsoons in East Asia and elevational reduction of eastern China since Mid-End Pleistocene [6,24]. Previous paleoclimates observation based on pollen data and δ^18^ Ο value demonstrated that the most substantial glacial extension occurred during the Marine Isotope Stages 16–18 (MIS 16-MIS 18, 0.6–0.7 Ma) in China [86]. After that, environmental changes seem to be moderate in subsequent climate oscillations in eastern China, where populations were growing stably throughout the glaciations.

### Secondary contact

Secondary contact of previously isolated populations in North America and Europe were well established [2,87]. In the present study, two divergent lineages were found to coexist at two localities (Fig 1). Haplotype SH-FNS3 representing four individuals from Funiushan Mts. (FNS) was nested into Lineage C, as well as two individuals of haplotype SH-SNJ24 from Shennongjia Mts. (SNJ) was resolved as a part of Lineage C, implying secondary contact after the initial divergence, caused by the expansion of Lineage C. Phylogenetic branching patterns have been used in studies on amphibians with limited dispersal capabilities to suggest location of origin of a population and dispersal direction [42]. In this study, a general trend of *O*. *schmackeri* populations in Lineage C dispersing northward to the two localities SNJ and FNS may be suggested by the phylogenetic tree.

### Taxonomic implications

In this study, seven divergent evolutionary lineages have been identified in *O*. *schmackeri* species complex by analyses of the mitochondrial ND2+tRNA sequences, with mean pairwise (K2P) sequence distances from 7.8% to 21.1%, except a closer genetic distance (3.4%) between Lineage C and Lineage D. The seven-clade divergence is also supported by AMOVA (Table 1). The ND2 distances between all seven lineages are comparable to distances between other recognized amphibian and reptile sister species, which may vary from 7% to 15% [88,89]. Additionally, isolation of 3–7 Ma and adaptation to different environmental factors can provide a favourable environment for speciation.

All together, this information suggests that these taxa represent seven good lineage-based species (Fig 2). Clade A includes the type locality of *O*. *huanggangensis*, so it could be called *O*. *huanggangensis* lineage. The haplotypes of *O*. *yizhangensis* from Nanling did not form a monophyletic group on gene tree or networks, instead they were nested into Clade A. The most prominent diagnostic characters of *O*. *yizhangensis* are smooth head and back, bright green colour, with irregular dark large spots; tibio-tarsal articulation reaching the tip of snout when leg stretched forward [90]. These putative diagnostic characters are possibly a habitat-related polymorphism, and more investigations are needed to evaluate the nature of the morphological differences. Based on our genetic analyses, we suggest that *O*. *yizhangensis* would be considered a junior synonym of *O*. *huanggangensis*. Clade B, including the type locality of *O*. *tianmuii*, could be called *O*. *tianmuii* lineage. Based on our mitochondrial analyses, the main distribution area of the currently recognized species *O*. *tianmuii* is located in the basin of the Qiantang River. Clade C, containing the type locality of *O*. *schmackeri*, could be called the true *O*. *schmackeri* lineage. So the *O*. *schmackeri sensu stricto *occupies a more narrow distribution area such as the northern Hunan and southern Hubei. Clade D-G represent four additional cryptic species occurring in the Yangtze River basin.

However, compared with the general definitions of candidate species [91], this speculation may be confused by the genetic admixture between lineage C and F in Funiushan Mts. (FNS) and between lineage C and G in Shennongjia Mts. (SNJ). In addition, sympatric occurrence with admixture may be caused by introgressive hybridization between closely related species, which has been documented in some anuran species [29,92]. Additional ecological, behavioral and quantitative morphological approaches are needed so as to confirm the taxonomic delineations suggested herein.

### Conservation implications

Our study has provided a means for assessing the evolutionary distinctiveness of populations of *O*. *schmackeri* species complex that may need conservation actions. Our data might be useful to establish management units (MUs) and/or evolutionary significant units (ESUs), two commonly used designations for endangered taxa [93]. Management units are defined by either reciprocal monophyly in mtDNA or substantial allele frequency divergence at nuclear loci; ESUs are defined by the presence of both [93]. Considering these criteria, the seven lineages (A-G) within *O*. *schmackeri* species complex would be considered at least MUs. These seven regional populations may represent important components in the evolutionary and adaptive structure of the species complex, and thus any conservation policy should concentrate on protecting these regional populations.

## Supporting Information

S1 Table Summary of sample site details for *O*. *schmackeri* species complex. For each population sampled, geographic origin, identified lineage, number of haplotypes (*h*), sample size (*N*) and coordinates (longitude/latitude) are given. Haplotype diversity (*Hd*) and nucleotide diversity (*π*) for each population with sample size > 3 are presented.
(DOC)

S2 Table Haplotypes of the mtDNA ND2+ tRNA genes for *O*. *schmackeri* species complex. Sample size (*N*), “SH” represents shared haplotype, and the other codes are abbreviations for geographical locations corresponding to S1 Table.
(DOC)

## References

[1] Hewitt GM (1996) Some genetic consequences of ice ages, and their role in divergence and speciation. Biol J Linn Soc 58: 247–276.

[2] Hewitt G (2000) The genetic legacy of the Quaternary ice ages. Nature 405: 907–913. 10879524 10.1038/35016000

[3] Avise JC (2000) Phylogeography: The history and formation of species Massachusetts: Harvard University Press.

[4] Myers N, Mittermeier RA, Mittermeier CG, da Fonseca GAB, Kent J (2000) Biodiversity hotspots for conservation priorities. Nature 403: 854–858. 10.1038/35002501 10706275

[5] Qian H, Ricklefs RE (2000) Large-scale processes and the Asian bias in temperate plant species diversity. Nature 407: 180–182. 11001054 10.1038/35025052

[6] Zhang RZ (2002) Geological events and mammalian distribution in China. Acta Zool Sinica 48: 141–153.

[7] Huang S, He SP, Peng ZG, Zhao K, Zhao EM (2007) Molecular phylogeography of endangered sharp-snouted pitviper (*Deinagkistrodon acutus*; Reptilia, Viperidae) in Mainland China. Mol Phylogenet Evol 44: 942–952. 17643319 10.1016/j.ympev.2007.05.019

[8] Ding LI, Gan XN, He SP, Zhao EM (2011) A phylogeographic, demographic and historical analysis of the short-tailed pit viper (*Gloydius brevicaudus*): evidence for early divergence and late expansion during the Pleistocene. Mol Ecol 20: 1905–1922. 10.1111/j.1365-294X.2011.05060.x 21438932

[9] Zhong J, Liu ZQ, Wang YQ (2008) Phylogeography of the rice frog, *Fejervarya multistriata* (Anura: Ranidae), from China based on mtDNA D-loop sequences. Zool Sci 25: 811–820. 10.2108/zsj.25.811 18795815

[10] Yan F, Zhou W, Zhao H, Yuan Z, Wang Y, Jiang K, et al (2013) Geological events play a larger role than Pleistocene climatic fluctuations in driving the genetic structure of *Quasipaa boulengeri* (Anura: Dicroglossidae). Mol Ecol 22: 1120–1133. 10.1111/mec.12153 23216961

[11] Clark MK, Schoenbohm LM, Royden LH, Whipple KX, Burchfiel BC, Zhang X, et al (2004) Surface uplift, tectonics, and erosion of eastern Tibet from large-scale drainage patterns. Tectonics 23: 1–20.

[12] An ZS, Kutzbach JE, Prell WL, Porter SC (2001) Evolution of Asian monsoons and phased uplift of the Himalaya-Tibetan plateau since Late Miocene times. Nature 411: 62–66. 11333976 10.1038/35075035

[13] Li JJ, Xie SY, Kuang MS (2001) Geomorphic evolution of the Yangtze Gorges and the time of their formation. Geomorphology 41: 125–135.

[14] An ZS, Zhang PZ, Wang EQ, Wang SM, Qiang XK, Li L, et al (2006) Changes of the monsoon-arid environment in China and growth of the Tibetan plateau since the Miocene. Quat Sci 26: 678–693.

[15] Zhang RZ (2004) Relict distribution of land vertebrates and Quaternary glaciation in China. Acta Zool Sinica 50: 841–851.

[16] Ju LX, Wang HJ, Jiang DB (2007) Simulation of the last glacial maximum climate over East Asia with a regional climate model nested in a general circulation model. Palaeogeogr Palaeocl 248: 376–390.

[17] Avise JC, Walker D (1998) Pleistocene phylogeographic effects on avian populations and the speciation process. Proc Roy Soc B-Biol Sci 265: 457–463. 10.1098/rspb.1998.0317 PMC1688909 9569664

[18] Knowles LL (2000) Tests of Pleistocene speciation in montane grasshoppers (genus *Melanoplus*) from the sky islands of western North America. Evolution 54: 1337–1348. 11005300 10.1111/j.0014-3820.2000.tb00566.x

[19] Starkey DE, Shaffer HB, Burke RL, Forstner MR, Iverson JB, Janzen FJ, et al (2003) Molecular systematics, phylogeography, and the effects of Pleistocene glaciation in the painted turtle (*Chrysemys picta*) complex. Evolution 57: 119–128. 12643572 10.1111/j.0014-3820.2003.tb00220.x

[20] Smith CI, Farrell BD (2005) Phylogeography of the longhorn cactus beetle *Moneilema appressum* LeConte (Coleoptera: Cerambycidae): was the differentiation of the Madrean sky islands driven by Pleistocene climate changes? Mol Ecol 14: 3049–3065. 16101773 10.1111/j.1365-294X.2005.02647.x

[21] Ursenbacher S, Conelli A, Golay P, Monney JC, Zuffi MAL, Thiery G, et al (2006) Phylogeography of the asp viper (*Vipera aspis*) inferred from mitochondrial DNA sequence data: evidence for multiple Mediterranean refugial areas. Mol Phylogenet Evol 38: 546–552. 16213755 10.1016/j.ympev.2005.08.004

[22] Miraldo A, Hewitt GM, Paulo OS, Emerson BC (2011) Phylogeography and demographic history of *Lacerta lepida* in the Iberian Peninsula: multiple refugia, range expansions and secondary contact zones. BMC Evol Biol 11: 170 10.1186/1471-2148-11-170 21682856 PMC3141430

[23] Guest HJ, Allen GA (2014) Geographical origins of North American Rhodiola (Crassulaceae) and phylogeography of the western roseroot, *Rhodiola integrifolia* . J Biogeogr 41: 1070–1080.

[24] Liu DS, Li ZG (1996) Geography of Asia. Beijing: Commercial Press.

[25] Li SH, Yeung CKL, Feinstein J, Han L, Le MH, WANG CX, et al (2009) Sailing through the Late Pleistocene: unusual historical demography of an East Asian endemic, the Chinese Hwamei (*Leucodioptron canorum canorum*), during the last glacial period. Mol Ecol 18: 622–633. 10.1111/j.1365-294X.2008.04028.x 19215583

[26] Song G, Qu Y, Yin Z, Li S, Liu N, Lei F (2009) Phylogeography of the *Alcippe morrisonia* (Aves: Timaliidae): long population history beyond late Pleistocene glaciations. BMC Evol Biol 9: 143 10.1186/1471-2148-9-143 19558699 PMC2714695

[27] Axelrod DL, Al-Shehbaz I, Raven PH (1996) History of the modern flora of China In: Zhang A, Wu S, eds. Floristic characteristics and diversity of East Asian plants. New York: Springer.

[28] Jin Y, Brown RP, Liu N (2008) Cladogenesis and phylogeography of the lizard *Phrynocephalus vlangalii* (Agamidae) on the Tibetan Plateau. Mol Ecol 17: 1971–1982. 10.1111/j.1365-294X.2008.03721.x 18363665

[29] Chen W, Bi K, Fu JZ (2009) Frequent mitochondrial gene introgression among high elevation Tibetan megophryid frogs revealed by conflicting gene genealogies. Mol Ecol 18: 2856–2876. 10.1111/j.1365-294X.2009.04258.x 19500253

[30] Wang LY, Abbott RJ, Zheng W, Chen P, Wang Y, Liu J (2009) History and evolution of alpine plants endemic to the Qinghai-Tibetan Plateau: *Aconitum gymnandrum* (Ranunculaceae). Mol Ecol 18: 709–721. 10.1111/j.1365-294X.2008.04055.x 19175501

[31] Qu Y, Lei F, Zhang R, Lu X (2010) Comparative phylogeography of five avian species: implications for Pleistocene evolutionary history in the Qinghai-Tibetan plateau. Mol Ecol 19: 338–351. 10.1111/j.1365-294X.2009.04445.x 20002586

[32] Zhan X, Zheng Y, Wei F, Bruford MW, Jia C (2011) Molecular evidence for Pleistocene refugia at the eastern edge of the Tibetan Plateau. Mol Ecol 20: 3014–3026. 10.1111/j.1365-294X.2011.05144.x 21689184

[33] Lu B, Zheng Y, Murphy RW, Zeng X (2012) Coalescence patterns of endemic Tibetan species of stream salamanders (Hynobiidae: *Batrachuperus*). Mol Ecol 21: 3308–3324. 10.1111/j.1365-294X.2012.05606.x 22571598

[34] Zhou WW, Wen Y, Fu JZ, Xu YB, Jin JQ, et al (2012) Speciation in the *Rana chensinensis* species complex and its relationship to the uplift of the Qinghai-Tibetan Plateau. Mol Ecol 21: 960–973. 10.1111/j.1365-294X.2011.05411.x 22221323

[35] Zhou WW, Yan F, Fu JZ, Wu SF, Murphy RW, Che J, et al (2013) River islands, refugia and genetic structuring in the endemic brown frog *Rana kukunoris* (Anura, Ranidae) of the Qinghai-Tibetan Plateau. Mol Ecol 22: 130–142. 10.1111/mec.12087 23116243

[36] Zhang DR, Chen MY, Murphy RW, Che J, PANG JF, HU JS, et al (2010) Genealogy and palaeodrainage basins in Yunnan Province: phylogeography of the Yunnan spiny frog, *Nanorana yunnanensis* (Dicroglossidae). Mol Ecol 19: 3406–3420. 10.1111/j.1365-294X.2010.04747.x 20666999

[37] Zhang M, Rao D, Yang J, Yu G, Wilkinson J (2010) A Molecular phylogeography and population structure of a mid-elevation montane frog *Leptobrachium ailaonicum* in a fragmented habitat of southwest China. Mol Phylogenet Evol 54: 47–58. 10.1016/j.ympev.2009.10.019 19850143

[38] Li Z, Yu G, Rao D, Yang J (2012) Phylogeography and demographic history of *Babina pleuraden* (Anura, Ranidae) in southwestern China. PloS ONE 7: e34013 10.1371/journal.pone.0034013 22448286 PMC3309021

[39] Yu G, Zhang M, Rao D, Yang J (2013) Effect of pleistocene climatic oscillations on the phylogeography and demography of red knobby newt (*Tylototriton shanjing*) from Southwestern China. PloS ONE 8: e56066 10.1371/journal.pone.0056066 23424644 PMC3570421

[40] Wang B, Jiang J, Xie F, Li C (2012) Postglacial colonization of the Qinling Mountains: phylogeography of the Swelled Vent frog (*Feirana quadranus*). PloS ONE 7: e41579 10.1371/journal.pone.0041579 22848532 PMC3405020

[41] Wang B, Jiang J, Xie F, Li C (2013) Phylogeographic Patterns of mtDNA Variation Revealed Multiple Glacial Refugia for the Frog Species *Feirana taihangnica* Endemic to the Qinling Mountains. J Mol Evol 76: 112–128. 10.1007/s00239-013-9544-5 23381112

[42] Fu JZ, Weadick CJ, Zeng XM, Wang YZ, Liu ZJ, Zheng Y, et al (2005) Phylogeographic analysis of the *Bufo gargarizans* species complex: a revisit. Mol Phylogenet Evol 37: 202–213. 15869886 10.1016/j.ympev.2005.03.023

[43] Hu YL, Wu XB, Jiang ZG Yan P, Su X, Cao SY (2007) Population genetics and phylogeography of *Bufo gargarizans* in China. Biochem Genet 45: 697–711. 17879156 10.1007/s10528-007-9107-9

[44] Zhang H, Yan J, Zhang GQ, Zhou KY (2008) Phylogeography and demographic history of chinese black-spotted frog Populations (*Pelophylax nigromaculata*): Evidence for independent refugia expansion and secondary contact. BMC Evol Biol 8: 21 10.1186/1471-2148-8-21 18215328 PMC2262875

[45] Huang ZH, Liu NF, Liang W, Zhang YY, Liao XJ, Ruan L, et al (2010) Phylogeography of Chinese bamboo partridge, *Bambusicola thoracica thoracica* (Aves: Galliformes) in south China: Inference from mitochondrial DNA control-region sequences. Mol Phylogenet Evol 56: 273–280. 10.1016/j.ympev.2010.01.028 20132900

[46] Mao X, Zhu G, Zhang S, Rossiter SJ (2010) Pleistocene climatic cycling drives intra-specific diversification in the intermediate horseshoe bat (*Rhinolophus affinis*) in Southern China. Mol Ecol 19: 2754–2769. 10.1111/j.1365-294X.2010.04704.x 20561192

[47] Wu Y, Wang Y, Jiang K, Hanken J (2013) Significance of pre-Quaternary climate change for montane species diversity: Insights from Asian salamanders (Salamandridae: *Pachytriton*). Mol Phylogenet Evol 66: 380–390. 10.1016/j.ympev.2012.10.011 23110935

[48] Fei L, Hu SQ, Ye CY, Huang YZ (2009) Fauna sinica Amphibia, Anura, Ranidae (Vol 3). Beijing: Science Press.

[49] Frost DR (2014) Amphibian Species of the World: an Online Reference. Version 6.0. American Museum of Natural History, New York, USA Available: http://research.amnh.org/herpetology/amphibian/index.html.

[50] Chen XH, Chen Z, Jiang JP, Qiao L, Lu YQ, Zhou KY, et al (2013) Molecular phylogeny and diversification of the genus *Odorrana* (Amphibia, Anura, Ranidae) inferred from two mitochondrial genes. Mol Phylogenet Evol 69: 1196–1202. 10.1016/j.ympev.2013.07.023 23911727

[51] Sambrook J, Russell DW (2001) Molecular Cloning: A Laboratory Manual (3rd Edition). New York: Cold Spring Harbor Laboratory Press.

[52] Li YM, Zhang HB, Wu XY, Xue H, Yan P, Wu XB (2014) A novel mitogenomic rearrangement for *Odorrana schmackeri* (Anura: Ranidae) and phylogeny of Ranidae inferred from thirteen mitochondrial protein-coding genes. Amphibia-Reptilia 35: 331–343.

[53] Hall TA (1999) BIOEDIT: a user-friendly biological sequence alignment editor and analysis program for Windows 95/98/NT. In Nucleic acids symposium series 41: 95–98.

[54] Librado P, Rozas J (2009) DnaSP v5: A software for comprehensive analysis of DNA polymorphism data. Bioinformatics 25: 1451–1452. 10.1093/bioinformatics/btp187 19346325

[55] Swofford DL. (2002) PAUP*: Phylogenetic Analysis Using Parsimony (* and other methods) Version 4.0b10. Sunderland: Sinauer Associates.

[56] Posada D, Crandall KA (1998) Modeltest: testing the model of DNA substitution. Bioinformatics 14: 817–818. 9918953 10.1093/bioinformatics/14.9.817

[57] Ronquist F, Huelsenbeck JP (2003) MrBayes 3: Bayesian phylogenetic inference under mixed models. Bioinformatics 19: 1572–1574. 12912839 10.1093/bioinformatics/btg180

[58] Rambaut A, Drummond AJ (2007) Tracer Version 1.6. Available: http://beast.bio.edsac.uk/Tracer.

[59] Drummond AJ, Rambaut A (2007) BEAST: Bayesian evolutionary analysis by sampling trees. BMC Evol Biol 7: 214 17996036 10.1186/1471-2148-7-214 PMC2247476

[60] Beerli P, Hotz H, Uzzell T (1996) Geologically dated sea barriers calibrate a protein clock for Aegean water frogs. Evolution 50: 1676–1687. 28565728 10.1111/j.1558-5646.1996.tb03939.x

[61] Bossuyt F, Brown RM, Hillis DM, Cannatella DC, Milinkovitch MC (2006) Phylogeny and biogeography of a cosmopolitan frog radiation: Late Cretaceous diversification resulted in continent-scale endemism in the family Ranidae. Syst Biol 55: 579–594. 16857652 10.1080/10635150600812551

[62] Excoffier L, Smouse PE, Quattro JM (1992) Analysis of molecular variance inferred from metric distances among DNA haplotypes: application to human mitochondrial DNA restriction data. Genetics 131: 479–491. 1644282 10.1093/genetics/131.2.479 PMC1205020

[63] Excoffier L, Lischer HEL (2010) Arlequin suite ver 3.5: a new series of programs to perform population genetics analyses under Linux and Windows. Mol Ecol Resour 10: 564–567. 10.1111/j.1755-0998.2010.02847.x 21565059

[64] Kimura M (1980) A simple method for estimeting evolutionary rates of base substitutions through comparative studies of nucleotide sequence. J Mol Evol 16: 111–120. 7463489 10.1007/BF01731581

[65] Tamura K, Stecher G, Peterson D, Filipski A, Kumar S (2013) MEGA6: molecular evolutionary genetics analysis version 6.0. Mol Biol Evol 30: 2725–2729. 10.1093/molbev/mst197 24132122 PMC3840312

[66] Miller MP (1997) Tools for population genetic analyses (tfpga), 1.3: a Windows program for the analyses of allozyme and molecular population genetic data. Available: http://bioweb.usu.edu/mpmbio.

[67] Clement M, Posada D, Crandall KA (2000) TCS: a computer program to estimate gene genealogies. Mol Ecol 9: 1657–1660. 11050560 10.1046/j.1365-294x.2000.01020.x

[68] Pfenninger M, Posada D (2002) Phylogeographic history of the land snail *Candidula unifasciata* (Helicellinae, Stylommatophora): Fragmentation, corridor migration, and secondary contact. Evolution 56: 1776–1788. 12389722 10.1111/j.0014-3820.2002.tb00191.x

[69] Templeton AR, Boerwinkle E, Sing CF (1987) A cladistic analyses of phenotypic associations with haplotypes inferred from restriction endonuclease mapping. I. Basic theory and an analyses of Alcohol Dehydrogenase activity in Drosophila. Genetics 117: 343–351. 2822535 10.1093/genetics/117.2.343 PMC1203209

[70] Templeton AR, Sing CF (1993) A cladistic analyses of phenotypic associations with haplotypes inferred from restriction endonuclease mapping. IV. Nested analyses with cladogram uncertainty and recombination. Genetics 134: 659–669. 8100789 10.1093/genetics/134.2.659 PMC1205505

[71] Templeton AR, Routman E, Phillips CA (1995) Separating population structure from population history: a cladistic analysis of the geographical distribution of mitochondrial DNA haplotypes in the tiger salamander, *Ambystoma tigrinum* . Genetics 140: 767–782. 7498753 10.1093/genetics/140.2.767 PMC1206651

[72] Tajima F (1989) Statistical method for testing the neutral mutation hypothesis by DNA polymorphism. Genetics 123: 585–595. 2513255 10.1093/genetics/123.3.585 PMC1203831

[73] Fu YX (1997) Statistical tests of neutrality of mutations against population growth, hitchhiking and background selection. Genetics 147: 915–925. 9335623 10.1093/genetics/147.2.915 PMC1208208

[74] Rogers AR, Harpending H (1992) Population growth makes waves in the distribution of pairwise genetic differences. Mol Biol Evol 9: 552–569. 1316531 10.1093/oxfordjournals.molbev.a040727

[75] Crawford AJ (2003) Huge populations and old species of Costa Rican and Panamanian dirt frogs inferred from mitochondrial and nuclear gene sequences. Mol Ecol 12: 2525–2540. 12969459 10.1046/j.1365-294x.2003.01910.x

[76] Chen PJ (1995) Coastal mountains of southeast China, volcanism and desert climate during the Upper Cretaceous In: Sun A, Wang Y, editors. Sixth Symposium on Mesozoic Terrestrial Ecosystems and Biota. Beijing: China Ocean Press pp. 189–191.

[77] Yang JC (1993) Features and Evolution of Landforms in China. Beijing: China Ocean Press.

[78] Qu J, Liu N, Bao X, Wang X (2009) Phylogeography of the ring-necked pheasant (*Phasianus colchicus*) in China. Mol Phylogenet Evol 52: 125–132. 10.1016/j.ympev.2009.03.015 19328240

[79] Yan J, Wang Q, Chang Q, Ji X, Zhou K (2010) The divergence of two independent lineages of an endemic Chinese gecko, *Gekko swinhonis*, launched by the Qinling orogenic belt. Mol Ecol 19: 2490–2500. 10.1111/j.1365-294X.2010.04660.x 20636892

[80] Yang DY (2006) The Formation of Yangtze River Geomorphology. Beijing: The Geological Publishing House.

[81] Gamble T, Berendzen PB, Bradley Shaffer H, Starkey DE, Simons AM (2008) Species limits and phylogeography of North American cricket frogs (Acris: Hylidae). Mol Phylogenet Evol 48: 112–125. 10.1016/j.ympev.2008.03.015 18462953

[82] Wang CB, Wang T, Su YJ (2014) Phylogeography of *Cephalotaxus oliveri* (Cephalotaxaceae) in relation to habitat heterogeneity, physical barriers and the uplift of the Yungui Plateau. Mol Phylogenet Evol 80: 205–216. 10.1016/j.ympev.2014.08.015 25160902

[83] Wang J, Gao P, Kang M, Lowe AJ, Huang H (2009) Refugia within refugia: the case study of a canopy tree (*Eurycorymbus cavaleriei*) in subtropical China. J Biogeogr 36: 2156–2164.

[84] Hull J, Girman DJ (2005) Effects of Holocene climate change on the historical demography of migrating sharp-shinned hawks (*Accipiter striatus velox*) in North America. Mol Ecol 14: 159–170. 15643959 10.1111/j.1365-294X.2004.02366.x

[85] Fontanella FM, Feldman CR, Siddall ME (2008) Phylogeography of *Diadophis punctatus*: extensive lineage diversity and repeated patterns of historical demography in a transcontinental snake. Mol Phylogenet Evol 46: 1049–1070. 18055224 10.1016/j.ympev.2007.10.017

[86] Wu GJ, Pan BT, Guan QY, Gao HS (2002) The maximum glaciation and desert expansion in China during MIS16. J Glaciol Geocryol 24: 544–549.

[87] Zamudio KR, Savage WK (2003) Historical isolation, range expansion, and secondary contact of two highly divergent mitochondrial lineages in spotted salamanders (*Ambystoma maculatum*). Evolution 57: 1631–1652. 12940367 10.1554/02-342

[88] Garrigues T, Dauga C, Ferquel E, Choumet V, Failloux AB (2005) Molecular phylogeny of *Vipera Laurenti*, 1768 and the related genera *Macrovipera* (Reuss, 1927) and *Daboia* (Gray, 1842), with comments about neurotoxic *Vipera aspis aspis* populations. Mol Phylogenet Evol 35: 35–47. 15737580 10.1016/j.ympev.2004.11.004

[89] Oliver PM, Adams M, Lee MS, Hutchinson MN, Doughty P (2009) Cryptic diversity in vertebrates: molecular data double estimates of species diversity in a radiation of Australian lizards (Diplodactylus, Gekkota). Proc Roy Soc B-Biol Sci 276: 2001–2007. 10.1098/rspb.2008.1881 PMC2677245 19324781

[90] Fei L, Ye CY, Jiang JP (2007) A new frog of the Ranidae (Ranidae, Anura). Acta Zoot Sin 32: 989–992.

[91] Vieites DR, Wollenberg KC, Andreone F, Köhler J, Glaw F, et al (2009) Vast underestimation of Madagascar’s biodiversity evidenced by an integrative amphibian inventory. Proc Nat Acad Sci USA 106: 8267–8272. 10.1073/pnas.0810821106 19416818 PMC2688882

[92] Liu K, Wang F, Chen W, Tu L, Min MS, Bi K, et al (2010) Rampant historical mitochondrial genome introgression between two species of green pond frogs, *Pelophylax nigromaculatus* and *P*. *plancyi* . BMC Evol Biol 10: 201 10.1186/1471-2148-10-201 20587049 PMC2909235

[93] Moritz C (1994) Defining “evolutionary significant units” for conservation. Trends Ecol Evol 9: 373–375. 10.1016/0169-5347(94)90057-4 21236896

