# Supplementary material for: Vicariance and Its Impact on the Molecular Ecology of a Chinese Ranid Frog Species-Complex (Odorrana schmackeri, Ranidae)
Source: PLoS One. 2015 Sep 22;10(9):e0138757. doi: 10.1371/journal.pone.0138757 (PMC4578928; doi:10.1371/journal.pone.0138757)
Supplement: S1 Table — For each population sampled, geographic origin, identified lineage, number of haplotypes (h), sample size (N) and coordinates (longitude/latitude) are given. Haplotype diversity (Hd) and nucleotide diversity (π) for each population with sample size > 3 are presented. (DOC) [file pone.0138757.s001.doc]

| **Sample site** | **Locality** | **Code** | **Geographical coordinates** | **Altitude (m)** | **Lineage** | ***h*/*N*** | ***Hd*** | ***π*** |
| --- | --- | --- | --- | --- | --- | --- | --- | --- |
| 1 | Wuyishan, Wuyishan Co., Fujian Prov. | WYS | E117.8939°, N27.8792° | 430 | A | 5/24 | 0.638±0.061 | 0.00066±0.00053 |
| 2 | Daiyunshan, Dehua Co., Fujian Prov. | DYS | E118.1497°, N25.7211° | 823 | A | 1/1 | - | - |
| 3 | Yinpingshan, Dongguan City, Guangdong Prov. | YPS | E114.2344°, N22.8964° | 195 | A | 7/24 | 0.645±0.101 | 0.00124±0.00087 |
| 4 | Liuxihe, Conghua Co., Guangdong Prov. | LXH | E113.8344°, N23.7367° | 448 | A | 5/15 | 0.695±0.109 | 0.00095±0.00063 |
| 5 | Wuzhishan, Ruyuan Co., Guangdong Prov. | WZS | E113.0808°, N24.9219° | 486 | A | 3/13 | 0.154±0.126 | 0.00013±0.00028 |
| 6 | Nanling, Ruyuan Co., Guangdong Prov. | NL | E113.0164°, N24.9286° | 985 | A | 4/17 | 0.419±0.141 | 0.00040±0.00050 |
| 7 | Mangshan, Yizhang Co., Hunan Prov. | MS | E112.8340°, N24.9931° | 541 | A | 9/16 | 0.883±0.061 | 0.00168±0.00131 |
| 8 | Guposhan, Hezhou City, Guangxi Prov. | GPS | E111.5625°, N24.5958° | 476 | A | 3/7 | 0.524±0.209 | 0.00050±0.00055 |
| 9 | Maoershan, Xing’an Co., Guangxi Prov. | MES | E110.4699°, N25.7812° | 422 | A | 4/19 | 0.585±0.115 | 0.00084±0.00058 |
| 10 | Leigongshan, Leishan Co., Guizhou Prov. | LGS | E108.2681°, N26.4356° | 876 | A | 9/30 | 0.738±0.080 | 0.00084±0.00080 |
| 11 | Fanjingshan, Jiangkou Co., Guizhou Prov. | FJS | E108.7739°, N27.8456° | 525 | A | 6/33 | 0.509±0.102 | 0.00051±0.00056 |
| 12 | Nanxijiang, Yongjia Co., Zhejiang Prov. | NXJ | E120.7353°, N28.3056° | 67 | B | 6/14 | 0.769±0.089 | 0.00101±0.00087 |
| 13 | Tianmushan, Lin’an Co., Zhejiang Prov. | TMS | E119.4419°, N30.3142° | 362 | B | 5/22 | 0.528±0.118 | 0.00180±0.00122 |
| 14 | Huangshan, Taiping Co., Anhui Prov. | HS | E118.2556°, N30.1306° | 150 | B | 1/4 | 0.000±0.000 | 0.00000±0.00000 |
| 15 | Guniujiang, Qimen Co., Anhui Prov. | GNJ | E117.4941°, N29.9833° | 186 | B | 5/30 | 0.414±0.111 | 0.00040±0.00058 |
| 16 | Sanchahe, Shitai Co., Anhui Prov. | SCH | E117.4926°, N30.1023° | 195 | B | 7/22 | 0.476±0.128 | 0.00202±0.00130 |
| 17 | Jiuhuashan, Chizhou City, Prov. | JHS | E117.5414°, N30.3081° | 519 | B | 5/19 | 0.591±0.118 | 0.00245±0.00121 |
| 18 | Gaojiayan, Yichang City, Hubei Prov. | GJY | E111.0336°, N30.6025° | 165 | C | 7/30 | 0.687±0.080 | 0.00091±0.00072 |
| 19 | Hupingshan, Shimen Co., Hunan Prov. | HPS | E110.8151°, N29.9286° | 550 | C | 9/35 | 0.452±0.105 | 0.00044±0.00076 |
| 20 | Lushan, Jiujiang City, Jiangxi Prov. | LS | E115.9356°, N29.5750° | 199 | D | 1/27 | 0.000±0.000 | 0.000000±0.00000 |
| 21 | Wugongshan, Luxi Co., Jiangxi Prov. | WGS | E114.1478°, N27.4756° | 569 | D | 3/6 | 0.600±0.215 | 0.00226±0.00135 |
| 22 | Kuankuoshui, Suiyang Co., Guizhou Prov. | KKS | E107.1583°, N28.1275° | 753 | E | 2/21 | 0.095±0.084 | 0.00008±0.00024 |
| 23 | Lengshuihe, Jinsha Co., Guizhou Prov. | LSH | E105.9989°, N27.5431° | 751 | E | 2/36 | 0.056±0.052 | 0.00005±0.00021 |
| 24 | Funiushan, Nanzhao Co., Henan Prov. | FNS | E112.1456°, N33.3361° | 472 | F/C | 4/10 | 0.778±0.091 | 0.07740±0.02104 |
| 25 | Shennongjia, Shennongjia Forest Region, Hubei Prov. | SNJ | E110.5858°, N31.3792° | 472 | G/C | 2/36 | 0.108±0.068 | 0.01444±0.00978 |
| **Total** | **25** | **-** | **-** | **-** | **-** | **94/511** | **0.945±0.004** | **0.10087±0.00882** |
