# Supplementary material for: Vicariance and Its Impact on the Molecular Ecology of a Chinese Ranid Frog Species-Complex (Odorrana schmackeri, Ranidae)
Source: PLoS One. 2015 Sep 22;10(9):e0138757. doi: 10.1371/journal.pone.0138757 (PMC4578928; doi:10.1371/journal.pone.0138757)
Supplement: S2 Table — Sample size (N), “SH” represents shared haplotype, and the other codes are abbreviations for geographical locations corresponding to S1 Table. (DOC) [file pone.0138757.s002.doc]

| **Haplotype** | ***N*** | **Sample codes** | **GenBank Accession No.** |
| --- | --- | --- | --- |
| SH-E1 | 68 | JHS01-03, JHS05-06, JHS10-14, JHS18-19, GNJ01-05, GNJ07-09, GNJ11-17, GNJ20, GNJ22-23, GNJ25-26, GNJ28-30, HS01-04, SCH01-06, SCH08-14, SCH20, TMS01-02, TMS06, TMS08, TMS11-18, TMS20-22 | KP167484 |
| SH-SCH17 | 2 | SCH17, SCH21 | KP167485 |
| SCH19 | 1 | SCH19 | KP167486 |
| SH-E2 | 3 | SCH18, JHS15, SCH07 | KP167487 |
| SCH22 | 1 | SCH22 | KP167488 |
| JHS9 | 1 | JHS09 | KP167489 |
| SH-E3 | 8 | JHS04, JHS08, JHS16, NXJ04, NXJ09-10, NXJ12, TMS19 | KP167490 |
| SH-E4 | 3 | JHS07, JHS17, SCH16 | KP167491 |
| DYS1 | 1 | DYS01 | KP167492 |
| SH-S1 | 38 | FJS01-02, FJS05, FJS09-11, FJS13-14, FJS16-22, FJS24-25, FJS27-29, FJS31-33, LGS01-02, LGS05, LGS09-17, LGS20, LGS28-29 | KP167493 |
| SH-FJS7 | 2 | FJS07, FJS26 | KP167494 |
| SH-FJS3 | 3 | FJS03-04, FJS30 | KP167495 |
| FJS12 | 1 | FJS12 | KP167496 |
| SH-FJS15 | 2 | FJS15、FJS23 | KP167497 |
| SH-FJS6 | 2 | FJS06、FJS08 | KP167498 |
| SH-S2 | 50 | GPS01-03, GPS06-07, MS04-05, MS10-11, MS14, NL01-02, NL04, NL07-15, NL17, WZS01-04, WZS06-13, WYS01, WYS06, WYS11-15, WYS17, WYS19, WYS21, WYS24, YPS05, YPS09, YPS15, YPS19 | KP167499 |
| GPS4 | 1 | GPS04 | KP167500 |
| SH-S3 | 4 | GPS05, MS01, MS03, MS16 | KP167501 |
| GNJ18 | 1 | GNJ18 | KP167502 |
| GNJ27 | 1 | GNJ27 | KP167503 |
| SH-GNJ10 | 2 | GNJ10, GNJ19 | KP167504 |
| SH-GNJ6 | 2 | GNJ06, GNJ24 | KP167505 |
| SH-E5 | 2 | GNJ21, SCH15 | KP167506 |
| SH-LGS21 | 2 | LGS21, LGS23 | KP167507 |
| SH-LGS7 | 3 | LGS07, LGS19, LGS27 | KP167508 |
| SH-LGS6 | 2 | LGS06, LGS24 | KP167509 |
| LGS4 | 1 | LGS04 | KP167510 |
| LGS30 | 1 | LGS30 | KP167511 |
| SH-LGS22 | 2 | LGS22, LGS25 | KP167512 |
| SH-LGS3 | 3 | LGS03, LGS18, LGS26 | KP167513 |
| LGS8 | 1 | LGS08 | KP167514 |
| SH-S4 | 9 | LXH01-03, LXH08, LXH10, LXH12, LXH14-15, NL03 | KP167515 |
| LXH9 | 1 | LXH09 | KP167516 |
| SH-LXH6 | 2 | LXH06-07 | KP167517 |
| LXH11 | 1 | LXH11 | KP167518 |
| SH-LXH4 | 3 | LXH04-05, LXH13 | KP167519 |
| MS15 | 1 | MS15 | KP167520 |
| MS12 | 1 | MS12 | KP167521 |
| MS8 | 1 | MS08 | KP167522 |
| MS7 | 1 | MS07 | KP167523 |
| SH-MS2 | 2 | MS02, MS13 | KP167524 |
| MS9 | 1 | MS09 | KP167525 |
| MS6 | 1 | MS06 | KP167526 |
| SH-MES1 | 12 | MES01-04, MES06-08, MES12, MES15-16, MES18-19 | KP167527 |
| SH-MES13 | 3 | MES13-14, MES17 | KP167528 |
| SH-MES9 | 2 | MES09-10 | KP167529 |
| SH-MES5 | 2 | MES05, MES11 | KP167530 |
| SH-S5 | 2 | NL16, WZS05 | KP167531 |
| SH-NL5 | 2 | NL05-06 | KP167532 |
| SH-NXJ1 | 6 | NXJ01-02, NXJ05-06, NXJ11, NXJ13 | KP167533 |
| NXJ14 | 1 | NXJ14 | KP167534 |
| NXJ3 | 1 | NXJ03 | KP167535 |
| NXJ7 | 1 | NXJ07 | KP167536 |
| NXJ8 | 1 | NXJ08 | KP167537 |
| SH-TMS7 | 2 | TMS07, TMS09 | KP167538 |
| SH-TMS3 | 3 | TMS03-05 | KP167539 |
| TMS10 | 1 | TMS10 | KP167540 |
| WYS9 | 1 | WYS09 | KP167541 |
| SH-WYS2 | 10 | WYS02, WYS04-05, WYS07-08, WYS10, WYS16, WYS18, WYS20, WYS22 | KP167542 |
| WYS23 | 1 | WYS23 | KP167543 |
| WYS3 | 1 | WYS03 | KP167544 |
| SH-YPS1 | 14 | YPS01-04, YPS07-08, YPS10-11, YPS13, YPS16, YPS21-24 | KP167545 |
| YPS6 | 1 | YPS06 | KP167546 |
| YPS14 | 1 | YPS14 | KP167547 |
| YPS12 | 1 | YPS12 | KP167548 |
| YPS18 | 1 | YPS18 | KP167549 |
| SH-YPS17 | 2 | YPS17, YPS20 | KP167550 |
| SH-C1 | 42 | GJY02-03, GJY05-06, GJY09-11, GJY14-16, GJY19, GJY22-24, GJY26, GJY29, HPS01, HPS03-06, HPS08-10, HPS12, HPS14-16, HPS18-19, HPS21-23, HPS25-26, HPS28, HPS30-35 | KP167551 |
| SH-GJY4 | 3 | GJY04, GJY08, GJY25 | KP167552 |
| GJY12 | 1 | GJY12 | KP167553 |
| SH-GJY7 | 3 | GJY07, GJY21, GJY28 | KP167554 |
| SH-GJY1 | 5 | GJY01, GJY13, GJY17, GJY18, GJY20 | KP167555 |
| GJY30 | 1 | GJY30 | KP167556 |
| GJY27 | 1 | GJY27 | KP167557 |
| HPS27 | 1 | HPS27 | KP167558 |
| HPS24 | 1 | HPS24 | KP167559 |
| SH-HPS11 | 2 | HPS11, HPS20 | KP167560 |
| HPS29 | 1 | HPS29 | KP167561 |
| HPS17 | 1 | HPS17 | KP167562 |
| HPS2 | 1 | HPS02 | KP167563 |
| HPS13 | 1 | HPS13 | KP167564 |
| HPS7 | 1 | HPS07 | KP167565 |
| SH-W1 | 55 | KKS01-14, KKS16-21, LSH01-34, LSH36 | KP167566 |
| KKS15 | 1 | KKS15 | KP167567 |
| LSH35 | 1 | LSH35 | KP167568 |
| SH-C2 | 28 | LS01-27, WGS06 | KP167569 |
| SH-SNJ1 | 34 | SNJ01-23, SNJ25-31, SNJ33-36 | KP167570 |
| SH-SNJ24 | 2 | SNJ24, SNJ32 | KP167571 |
| SH-FNS3 | 4 | FNS03-05, FNS09 | KP167572 |
| SH-FNS2 | 3 | FNS02, FNS06, FNS10 | KP167573 |
| FNS7 | 1 | FNS07 | KP167574 |
| SH-FNS1 | 2 | FNS01, FNS08 | KP167575 |
| SH-WGS1 | 4 | WGS01-02, WGS04-05 | KP167576 |
| WGS3 | 1 | WGS03 | KP167577 |
